# Pan-cancer analysis of homeodomain-containing gene C10 and its carcinogenesis in lung adenocarcinoma

**DOI:** 10.18632/aging.205348

**Published:** 2023-12-27

**Authors:** Xiangyuan Tan, Zhanzhan Li, Huayan Xie, Jiarong Chen, Jian Xiao, Yaofeng Zhi, Haixin Mo, Yanming Huang, Aibin Liu

**Affiliations:** 1 Department of Oncology, Xiangya Hospital, Central South University, Changsha 410008, Hunan, China; 2 Department of Anesthesiology, The First Affiliated Hospital of Jinan University, Guangzhou 510000, Heyuan, China; 3 Department of Oncology, Jiangmen Central Hospital, Jiangmen 529030, Guangdong, China; 4 Department of Geriatrics, Xiangya Hospital, Central South University, Changsha 410008, Hunan, China; 5 National Clinical Research Center for Geriatric Disorders, Xiangya Hospital, Central South University, Changsha 410008, Hunan, China; 6 Clinical Experimental Center, Jiangmen Key Laboratory of Clinical Biobanks and Translational Research, Jiangmen Central Hospital, Jiangmen 529030, Guangdong, China

**Keywords:** HOXC10, LUAD, VEGFA, MVD, CTC clusters

## Abstract

We found elevated homeodomain-containing gene C10 (HOXC10) showed dual roles in cancers’ prognosis. Some signal pathways associated with tumor were totally positively enriched in HOXC10 for whole cancers. On the contrary, Notch signaling, Wnt-beta catenin signaling, myogenesis, and Hedgehog signaling were almost negatively enriched in HOXC10. Some pathways showed dual roles such as Kras signaling, interferon gram and alpha response, IL6/JAK/STAT3, IL2/STAT5 signaling. HOXC10 was associated with tumor mutation burden and microsatellite instability. HOXC10 also was associated with tumor microenvironment and immune status. HOXC10 was negatively associated with immune score in most cancers except colon adenocarcinoma. The correlations of HOXC10 with immune-related genes presented dual roles in different cancers. Results from our clinical samples indicated that HOXC10 was an independent predictor for distant metastasis-free survival in lung adenocarcinoma (LUAD). Notably, the high levels of HOXC10 were positively correlated with the expression of angiogenic markers, vascular endothelial growth factor and microvessel density, and the number of CTC clusters. Our results demonstrated that aberrant expression happened in most cancers, which also affected the clinical prognosis and involved in progression via multiple signal pathways cancers. HOXC10 overexpression plays an important role in the aggression and metastasis in LUAD, which indicated a potential therapeutic target and an independent factor for the prognosis for LUAD patients.

## INTRODUCTION

Globally, cancer is a major public health problem, and the global cancer burden is predicted to continue to increase for at least the next 20 years, and the United Nations has set reducing the cancer burden as a long-term development goal [1]. In order to reduce the burden of cancer disease, researchers in the field of cancer have been committed to finding the risk factors of cancer occurrence, exploring effective treatment programs, and analyzing the molecular mechanisms involved [2]. However, despite great efforts, cancer prevention and treatment have remained intractable problems. Therefore, the search for potential molecular markers will provide new insights into the treatment of cancer.

The Homeobox (HOX) genes are highly conserved at the genomic level and make major contributions to cell differentiation and embryonic development [3, 4]. HOX genes are arranged in four different clusters (A, B, C, and D), which located on four chromosomes (7, 17, 12, and 2) in mammals [5]. In recent years, mounting evidences have indicated that the abnormal expression of HOX genes play a crucial role in the metastasis and progress of multiple cancers, for instance, breast, thyroid, glioma, lung, and gastric cancers, by regulating important processes such as invasion, signal transduction, and angiogenesis [6]. Zhu et al. reported that overexpressed HOXD9 significantly promoted the proliferation, invasion, and migration of gastric cancer cells by activating the transcription of RUFY3, and correlated with poor survival [7]. Shah et al. reported that HOXB13 mediates invasiveness in human ER-positive breast cancer [8]. HOXA9 silencing prevents invasive prostate cancer metastasis induced by Twist1 [9]. The high expression of HOXA10 promoted the proliferation, migration and invasion of lung adenocarcinoma cells, and reduced cell apoptosis [10]. These results indicated that HOX genes might provide potential targets for future tumor therapies. However, there is no systematic exploration of all 39 HOX genes expression with metastasis in lung adenocarcinoma.

As a member of the HOX gene family, HOXC10 belongs to the HOX gene family C cluster. Many studies have shown that abnormal expression of HOXC10 contributes to the malignant progression of several types of cancers. Dang and colleagues reported that overexpression of HOXC10 promotes hepatocellular carcinoma metastasis by transactivating PDPK1 and VASP expression [11]. Kim et al. found that overexpressed HOXC10 correlates with poor survival and recurrence in gastric cancer [12]. HOXC10 promotes metastasis by regulating the transcription of the EMT-related gene Slug in ovarian cancer [13]. Moreover, Tang et al. reported that high level of HOXC10 plays a vital role in migration, invasion and adhesion of lung cancer cells and indicates worse prognosis by analyzing HOXC10 expression in 63 clinical samples and public database [14]. However, the biological function and clinical role of HOXC10 in pan-cancer remains vague.

In this study, we first analyzed the differential expressions of HOXC10 in pan-cancer and corresponding normal samples, and explored its prognosis roles in cancers. Next, we investigated the pathways enrichment and molecular features. Then, we evaluated the effect of HOXC10 on tumor microenvironment and immune infiltration in cancers. Finally, we investigated the expression and clinical significance of HOXC10 in the LUAD datasets from The Cancer Genome Atlas (TCGA), and 408 LUAD samples and 46 benign lung disease samples. Our results found that HOXC10 was highly expressed in LUAD clinical samples, and its overexpression is significantly correlated with the pathological grade, clinical stage, N classification, and M classification, as well as poor distant metastasis-free survival (DMFS). Importantly, the high levels of HOXC10 were positively correlated with the expression of VEGFA and MVD, and the number of CTC clusters. Therefore, our results uncover that HOXC10 upregulation plays an important role in the aggression and metastasis in LUAD, which might be a potential target for treatment of lung cancer and an independent factor for the prognosis of LUAD.

## MATERIALS AND METHODS

### Patients and clinical specimens

The mRNA expression profile and clinical information of 33 types of cancers and normal tissues (eQTL: expression quantitative trait loci, https://www.gtexportal.org/home/) were extracted from The Cancer Genome Atlas data platform. We also downloaded the immune cells and immunophenotype scores from the ImmuCellAI. The clinicopathological features of the 46 patients with benign lung disease were summarized in Supplementary Table 1, which were obtained during surgery and the clinicopathological features of 408 LUAD specimens are summarized in Supplementary Table 2, which were obtained during surgery or needle biopsy. All tissues were obtained from January 2010 and December 2020 at Jiangmen Central Hospital (Guangdong, China). The diagnoses were based on clinical and pathological results. The use of these clinical materials for research purposes have obtained prior consent of patient and approval by the Institutional Research Ethics Committee of Jiangmen Central Hospital. Two independent professional pathologists assessed the ratio of tumor vs non-tumor of hematoxylin and eosin (H&E) stained tissue samples. More than 75% of the clinical lung cancer tissue samples analyzed in this study were used for further analysis.

### Bioinformatic analysis

In this study, the following bioinformatic analysis methods were used: (1) Differentially expressed genes analysis was performed using R “limma” packages; (2) Using the follow-up data and HOXC10 expression, we analyzed the correlations of HOXC10 with four clinical outcomes: overall survival (OS: the period from date of diagnosis until death from any case), disease specific survival (DSS: the percent of people who died from a specific disease in a defined period of time), disease free interval (DFI: the measure of time after treatment during which no sign of cancer is found), and progression free interval (PFI: the length of time during and after the treatment of a disease) [15]. The Kaplan-Meier survival curves were plotted, and the forest plot of the results of univariate Cox regression with hazard ratio (HR) and their 90% confidence interval (CIs); (3) Gene set variation analysis was performed to compare the differentially expressed pathways of different levels of HOXC10 expression using “GSVA” package; (4) we explored the correlations of HOXC10 with tumor mutation burden (TMB) and microsatellite instability (MSI) using Pearson correlation analysis. (4) We explored the tumor microenvironment by calculating ESTIMATE score, stromal score, and immune score. (5) Pearson correlation analysis was used to present the correlations of HOXC10 expression with 22 immune score, 22 kinds of immune cells and function, immune activated genes, immune inhibited genes, chemokines genes and chemokines receptors genes.

### Immunohistochemistry (IHC)

IHC analysis was conducted to examine HOXC10 expression in 46 benign lung disease and 408 LUAD samples, VEGFA and CD31 expression in 238 LUAD samples with pathological grade II. The IHC performance and expression scoring criterion were stated previously [16–18]. The formalin-fixed paraffin-embedded sections were boiled in TE (pH 9.0) buffer for 10 min to extract antigens, blocked with hydrogen peroxide and goat serum, then incubated overnight at 4° C in a humidified box with anti-HOXC10 antibody (1:100, Cat#PA5-31078, Invitrogen, Carlsbad, CA, USA), anti-VEGFA antibody (1:200, Cat#19003-1-AP, Proteintech, Wuhan, China), and anti-CD31 antibody (1:2000, Cat#11265-1-AP, Proteintech) in Antibody Diluent (Abcam, Cambridge, MA, USA). After incubation, slides were rinsed in TBS/0.05% Tween 20, incubated with biotin-conjugated secondary antibody (Proteintech) and horseradish peroxidase (HRP)-conjugated streptavidin (Proteintech) 30 min at room temperature, respectively, stained by 3,3′-Diaminobenzidine (DAB) Liquid Substrate Dropper System (Sigma-Aldrich, St Louis, MO, USA). Staining index (SI) evaluated by two experienced independent investigators was averaged for comparative evaluation for HOXC10 and VEGFA expression. SI was the product of the ratio of positive tumor cells and staining intensity score. Positive tumor cell ratio was scored as follows: 0 (no positive tumor cells); 1 (< 10% positive tumor cells); 2 (10-35% positive tumor cells); 3 (35-70% positive tumor cells) and 4 (> 70% positive tumor cells). Staining intensity score was according to the following criteria: 0 (no staining); 1 (weak staining, light yellow); 2 (moderate staining, yellow brown) and 3 (strong staining, brown). Based on this method, expressions of HOXC10 and VEGFA in LUAD samples were evaluated with SI scores of 0, 1, 2, 3, 4, 6, 8, 9 or 12. SI score 4 was the median of tumor tissues. Low and high expression of HOXC10 and VEGFA were stratified by the following standard: SI ≤ 4 was conducted tumor samples with low level of HOXC10 and VEGFA, and SI score of > 4 as tumors with high level of HOXC10 and VEGFA. M8 digital microscope (PreciPoint, Freising, Bavaria, Germany) was used to capture images of human lung tissue under 10× and 40× objective magnification.

### Microvascular density (MVD)

CD31 stained slides were examined to determine MVD in clinical samples. For each slide, 3 hot spot fields (the largest number of stained vessels) were evaluated under a 50× light microscope. The pathologist subsequently counts the blood vessels at each "hot spot" under a 200 × (high power) light microscope. The calculation of MVD of each hot spot was based on the total number of vessels divided by the area. The MVD of the specimen was used as the average of the MVD of the three histological regions. For statistical analysis, the MVD was divided into low (below the median) and high (greater than the median) groups [19].

### Subtraction enrichment (SE) of blood samples for CTCs

For blood samples, using Human Circulating Tumor Cell Subtraction Enrichment Kit (Cat#SHE-011, Cytelligen, SanDiego, CA, USA) enriched CTCs according to the manufactures’ direction, collecting 6 ml peripheral blood, and 200 ×g centrifugal for 15 minutes. 3.5ml hCTC buffer was used for softly mixing with the precipitated blood cells and subsequently loaded on nonhematopoietic cell separation matrix, then centrifuging at 450 × g for 5 min. The supernatant containing tumor cells was resuspended to 14 mL hCTC buffer. Samples were then rotated at room temperature (500 × g) for 4 min, then the supernatant is aspirated to 50 μl. Precipitated cells were softly resuspended and bleed with the cell fixative (Cytelligen, San Diego, CA, USA). Samples were painted on CTC slides, and dried overnight for subsequent immunostaining-fluorescence *in situ* hybridization analyses.

### Identification of CTCs using immunostaining-fluorescence *in situ* hybridization (iFISH)

iFISH was conducted according to the manufacturer’s guidance (Cytelligen). Dry monolayer-coated slides were incubated with PBS at room temperature for 3 min, followed by hybridized Vysis chromosome 8 centromere probe (CEP8) SpectrumOrange (Abbott Laboratories, Chicago, IL, USA) for 4 hours utilizing an S500 StatSpin ThermoBrite Slide Hybridization/Denaturation System (Abbott Molecular, Abbott Park, IL, USA). Subsequently, samples were hatched with Alexa Fluor (AF) 488 (green)-conjugated monoclonal anti-CD45 antibodies (1:200, Cat#FSH-002, Cytelligen, San Diego, CA, USA) for 30 min in the dark. Cell nucleus staining were performed with DAPI (Beyotime, Shanghai, China). CTC image scanning and analysis were used to obtain the recognized tumor cell image automatically. CTCs were defined as DAPI+, CD45-, CEP8 >2 signals.

### Statistical analysis

The chi-squared test was used to analyze the connection between HOXC10 expression and clinicopathological characteristics. For normal distributed variables, data are presented as the mean ± standard deviation (SD) and the statistical difference between the two groups was determined using the student’s t-test. The statistical differences between multiple groups were determined by one-way ANOVA. The median and interquartile range present continuous variables without normal distribution. Nonparametric test (Mann-Whitney U-test) was used to assess the significance of the differences. Survival curves for both HOXC10-high and HOXC10-low patients (median) were utilized by the Kaplan-Meier method, which were compared by the log-rank test. *P* < 0.05 indicated a statistically significant difference. All analyses were conducted by SPSS 23.0 software (IBM Corp, Chicago, IL, USA) and diagrams were produced by GraphPad Prism 8.0 software (GraphPad Inc, San Diego, CA, USA).

## RESULTS

### Differential expression of HOXC10 and its associations with prognosis in pan-cancer

**Figure 1:**
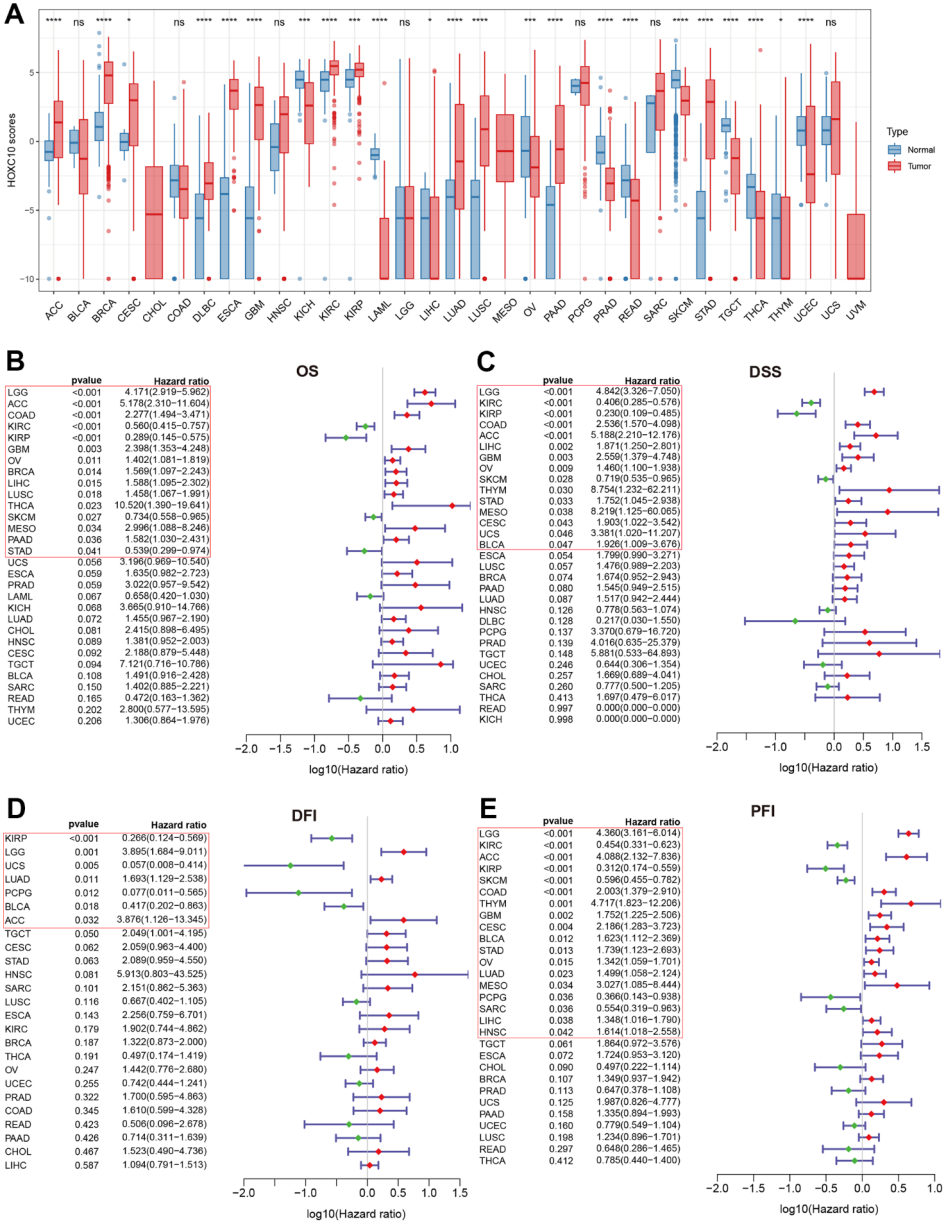
**Abnormal expression of HOXC10 and its relevance with clinical prognosis.** (**A**) Expression levels of HOXC10 between tumor and normal tissues. (**B**–**E**) Forest plot showed the correlations of HOXC10 with clinical prognosis by univariate Cox regression: OS, DSS, PFI and DFI.

We first evaluated the expression levels of HOXC10 between normal and tumor pan-cancer tissues. The result indicated that HOXC10 was significantly up-regulated in most cancers including BRCA, CESC, DLBC, ESCA, GBM, KIRC, KIRP, LUAD, LUSC, PAAD, SARC, STAD, and THYM (Figure 1A). However, the expression of HOXC10 was remarkably down-regulated in KICH, LAML, OV, PRAD, READ, SKCM, TGCT, THCA, UCEC (Figure 1A). Next, we analyzed the associations between HOXC10 expression and four clinical outcomes. Our results shown that elevated HOXC10 was associated with poor OS in LGG, ACC, COAD, GBM, OV, BRCA, LIHC, LUSC, THCA, MESO, and PAAD but was negatively associated with OS in KIRC, KIRP, SKCM, and STAD (Figure 1B). The Kaplan-Meier survival curve also shown same trends, and indicated high-expressed HOXC10 was associated with poor OS in PRAD and UCS (Supplementary Figure 1). Similarly, our results suggested that the high expression of HOXC10 was positively associated with poor DSS in LGG, COAD, ASS, LIHC, GBM, OV, THYM, STAD, MESO, CESC, UCS, and BLCA. But favorable DSS was found for patients with elevated HOXC10 expression in KIRC, KIRP and SKCM (Figure 1C). The Kaplan-Meier survival curve identified unfavorable and favorable roles in these cancers (Supplementary Figure 2). For DFI, high-expressed HOXC10 increased the risk of poor prognosis in LGG, LUAD, and ACC but decreased the risk of poor prognosis in KIRP, UCS, PCPG, and BLCA (Figure 1D). The Kaplan-Meier survival curve also suggested that elevated increased the risk of poor DSS in HNSC, MESO, and TGCT (Supplementary Figure 3). Results for PFI indicated that elevated-HOXC10 was associated with poor PFI in LGG, ACC, COAD, THYM, GBM, CESC, BLCA, STAD, OV, LUAD, MESO, LIHC, and HNSC but was favorable for DSS in KIRC, KIRP, SKCM, PCPG, and SARC (Figure 1E). Same trends were also found in Kaplan-Meier analyses (Supplementary Figure 4).

### Pathways enrichment and molecular features of HOXC10 in pan-cancer

**Figure 2:**
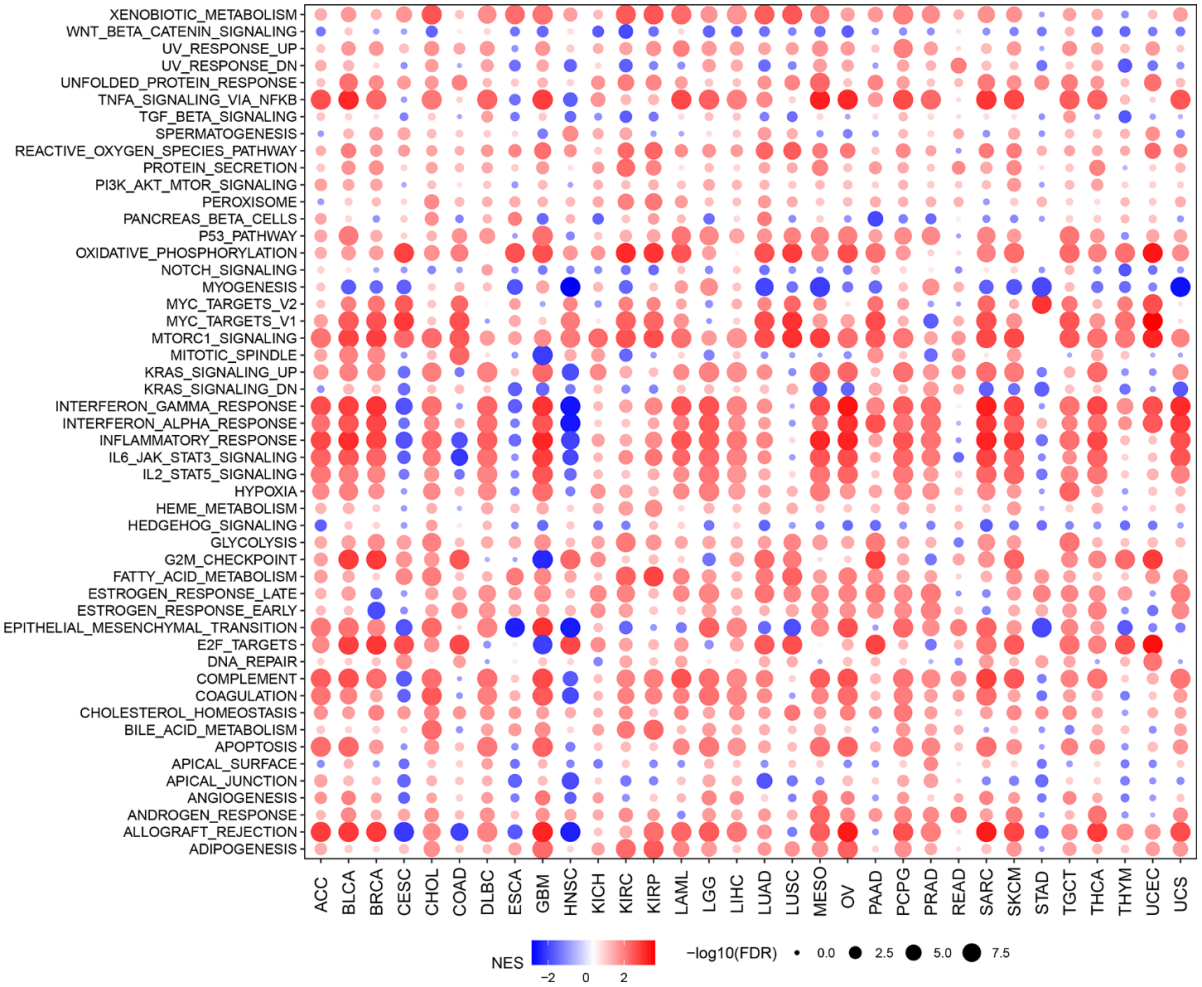
GSVA indicated the associations between HOXC10 expression and signaling pathways.

To explore the biological function roles of HOXC10 in cancers, we analyzed the correlations of the pathway’s enrichment differences with HOXC10 expression (Figure 2). We found TNFA signaling via NFKB, reactive oxygen species pathway, PI3K/AKT/MTOR signaling, P53 pathway, oxidative phosphorylation, MTORC1 signaling, glycolysis, fatty acid metabolism, hypoxia and apoptosis were totally positively enriched in HOXC10 for whole cancers. On the contrary, Notch signaling, Wnt-beta catenin signaling, myogenesis, and Hedgehog signaling were almost negatively enriched in HOXC10. Some pathways showed dual roles such as Kras signaling, interferon gram and alpha response, IL6/JAK/STAT3, IL2/STAT5 signaling were negatively enriched in HOXC10 for CESC, COAD, ESCA, HNSC but were positively enriched in HOXC10 for other cancers. Some immune-related pathways such as apical surface and junction, angiogenesis, and allograft rejection were also negatively enriched in CESC, ESCA, and HNSC.

**Figure 3:**
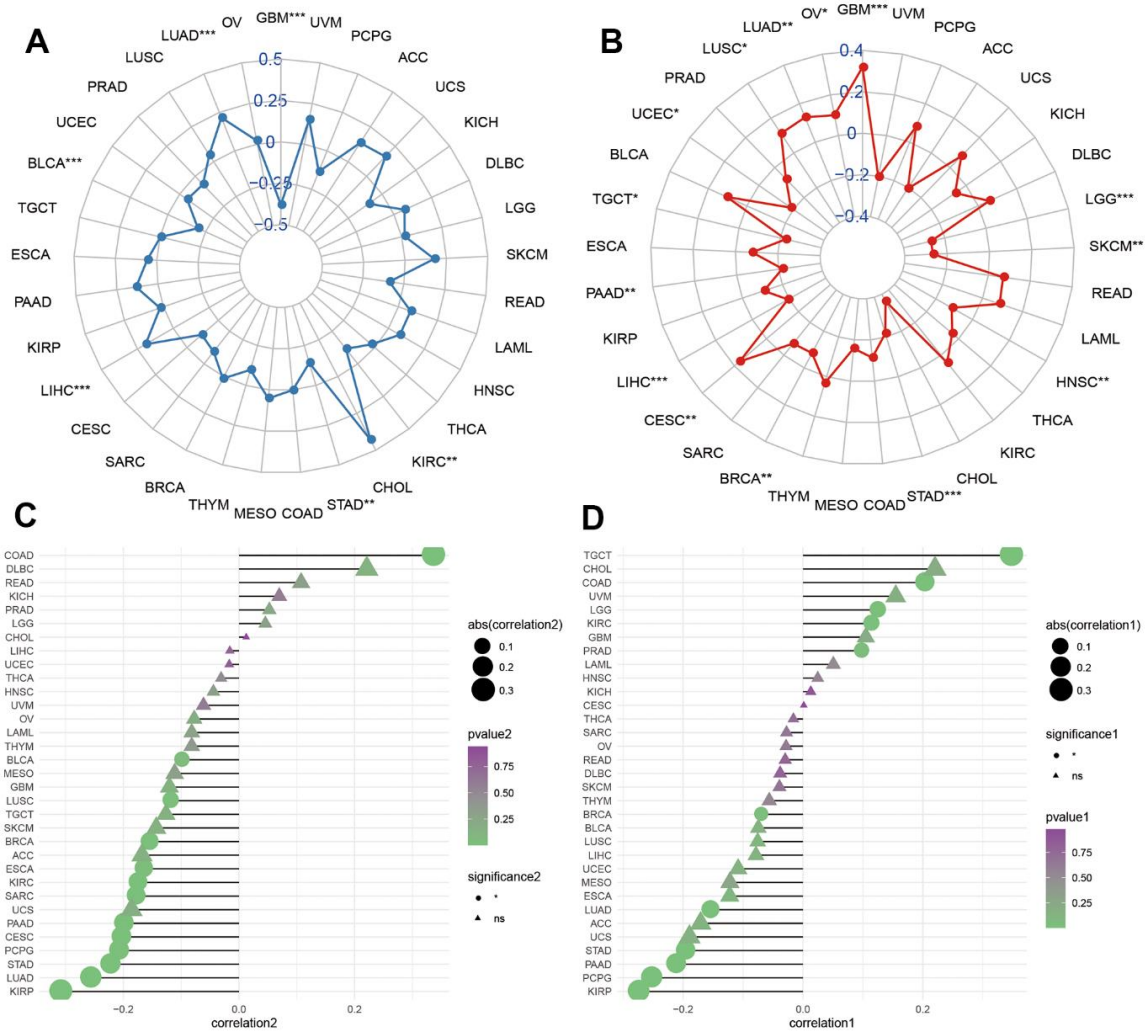
**Molecular features of HOXC10 in cancers.** (**A**, **B**) Correlations of HOXC10 with TMB and MSI in cancers. (**C**, **D**) Correlations of HOXC10 with ESTIMATE and stromal score.

We further explored the correlations of HOXC10 with TMB and MSI. We found that HOXC10 was positively associated with TMB in LIHC, LUAD and was negatively associated with TMB in GBM, KIRC, STAD, and BLCA (Figure 3A). While HOXC10 showed a positively connection with MSI in GBM, LGG, CESC, LUSC, LUAD, and OV but a negative correlation to MSI in SKCM, STAD, BRCA, LIHC, PAAD, TGCT, and UCEC (Figure 3B).

We estimated the correlations of HOXC10 with tumor microenvironment using ESTIMATE and stromal scores. For KIRP, LUAD, STAD, PCPG, CESC, PAAD, SARC, KIRC, ESCA, BRCA, LUSC, and BLCA, the expression of HOXC10 was negatively associated with ESTIMATE score while showed a positive connection only for COAD. For stromal score, negative associations between HOXC10 expression and stromal score were found in KIRP, PCPG, PAAD, STAD, LUAD, BRCA while HOXC10 presented positive connections with stromal score for TGCT, COAD, LGG, KIRC, and PRAD.

### Association between HOXC10 and immune status in pan-cancer

**Figure 4:**
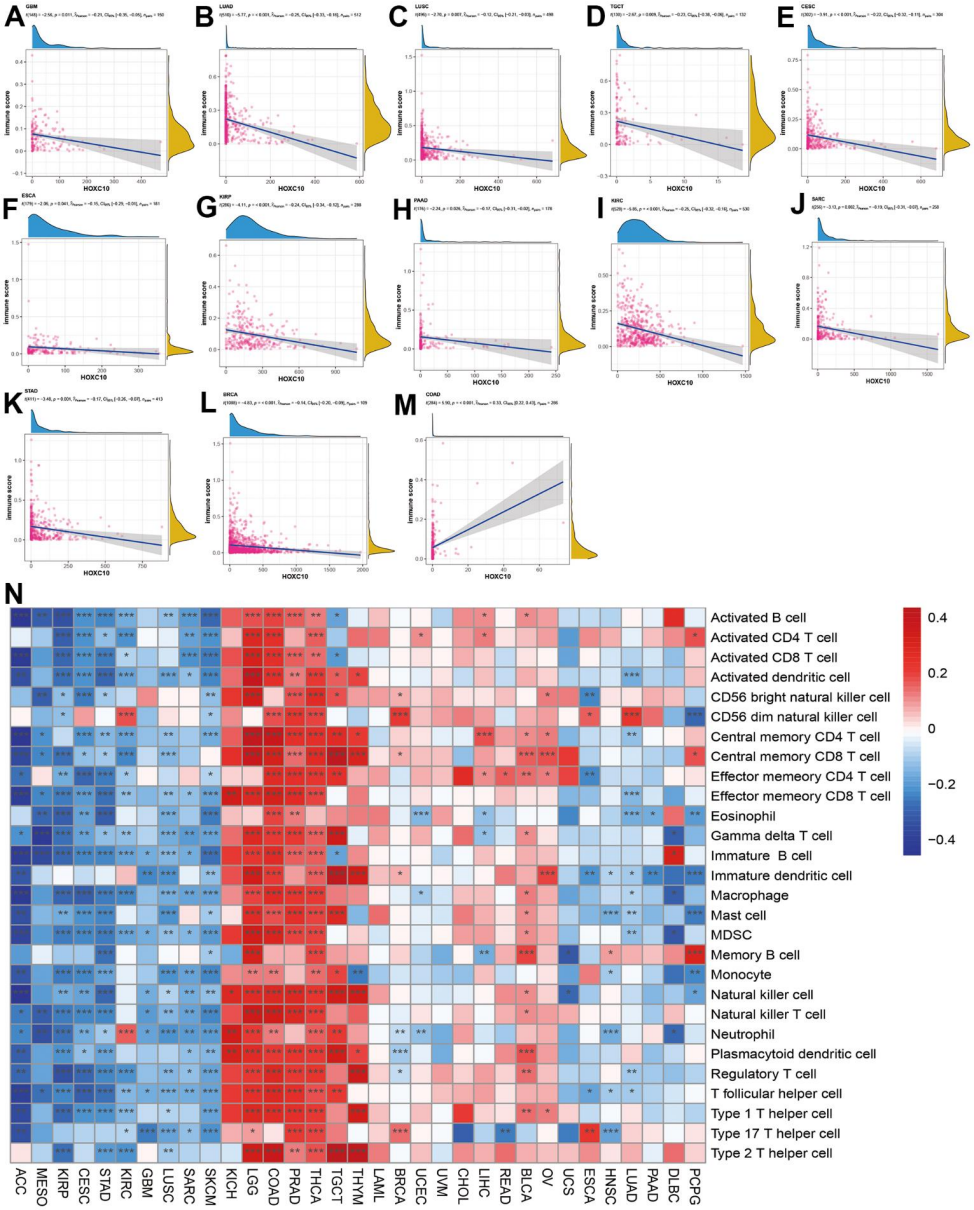
**Associations between HOXC10 expression and immune status.** (**A**–**M**) Correlations of HOXC10 with immune score in GBM, LUAD, LUSC, TGCT, CESC, ESCA, KIRP, PAAD, KIRC, SARC, STAD, BRCA, and COAD. (**N**) Associations between HOXC10 and immune cells in cancers.

To explore the immune regulation role of HOXC10 in cancers, we analyzed the associations between HOXC10 expression and immune score and immune-related genes. Our results showed that HOXC10 was negatively associated with immune score in GBM, LUAD, LUSC, TGCT, CESC, ESCA, KIRP, PAAD, KIRC, SARC, STAD, and BRCA (Figure 4A–4L). However, a positive connection was found between HOXC10 expression and immune score in COAD.

We next evaluated the correlations of HOXC10 with immune infiltration in cancers. Our results indicated that HOXC10 was almost positively correlated with all immune cells infiltration levels in KICH, LGG, COAD, PRAD, THCA, TGCT and THYM. On the contrary, these immune cells negatively infiltrated with HOXC10 in ACC, MESO, KIRP, CESC, STAD, GBM, LUSC, SARC, and SKCM. Only, neutrophil and CD56 dim natural killer cell were positively with HOXC10 in KIRC. In LUAD and HNSC, immune cells show similar trends (Figure 4N).

**Figure 5:**
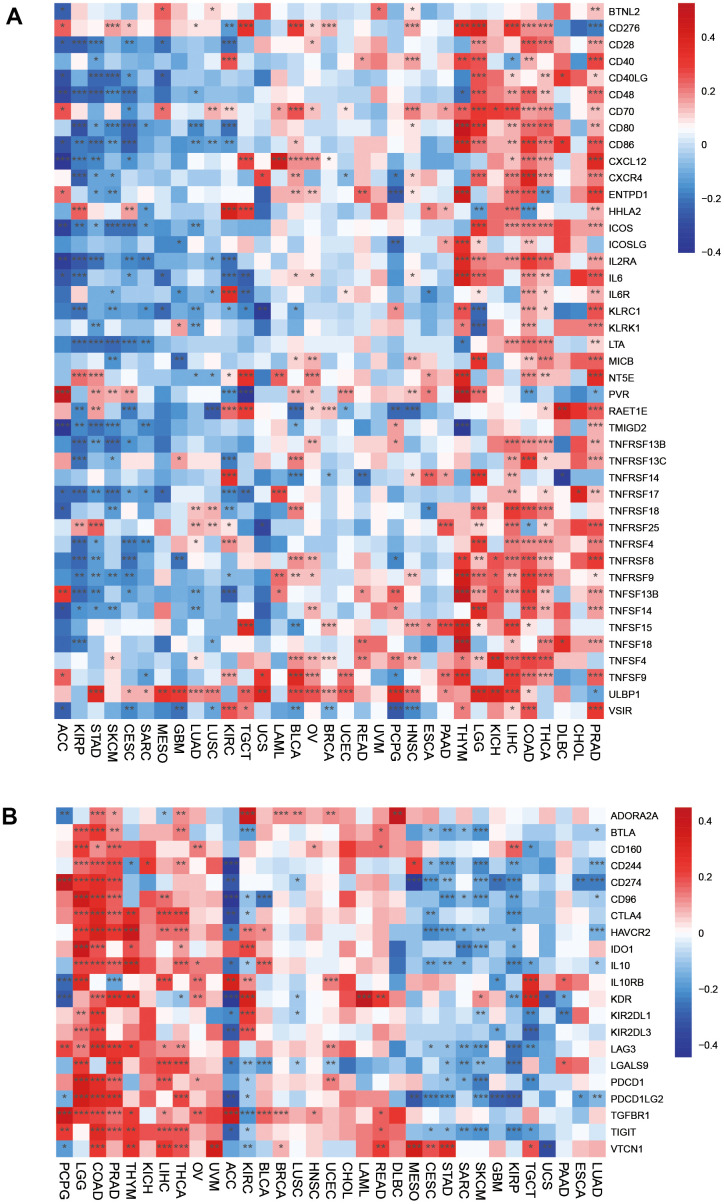
**Associations between HOXC10 and immune-related genes.** (**A**) Immune-activated genes. (**B**) Immune-inhibited genes.

Immune-activated genes were positively associated with HOXC10 expression in THYM, LGG, KICH, LIHC, COAD, THCA, DLBC, CHOL, and PRAD. However, HOXC10 seems to inhibit these immune genes expression in ACC, KIRP, STAD, SKCM, CESC, SARC, MESO, GBM, LUAD, LUSC, KIRCT, TGCT, and UCS. For immune-inhibited genes, HOXC10 was positively associated with these genes such as CD274 in PCPG, LGG, COAD, PRAD but showed negative with CD274 in CESC, STAD, SKCM, GBM, KIRP, ESCA, and LUAD (Figure 5A, 5B).

**Figure 6:**
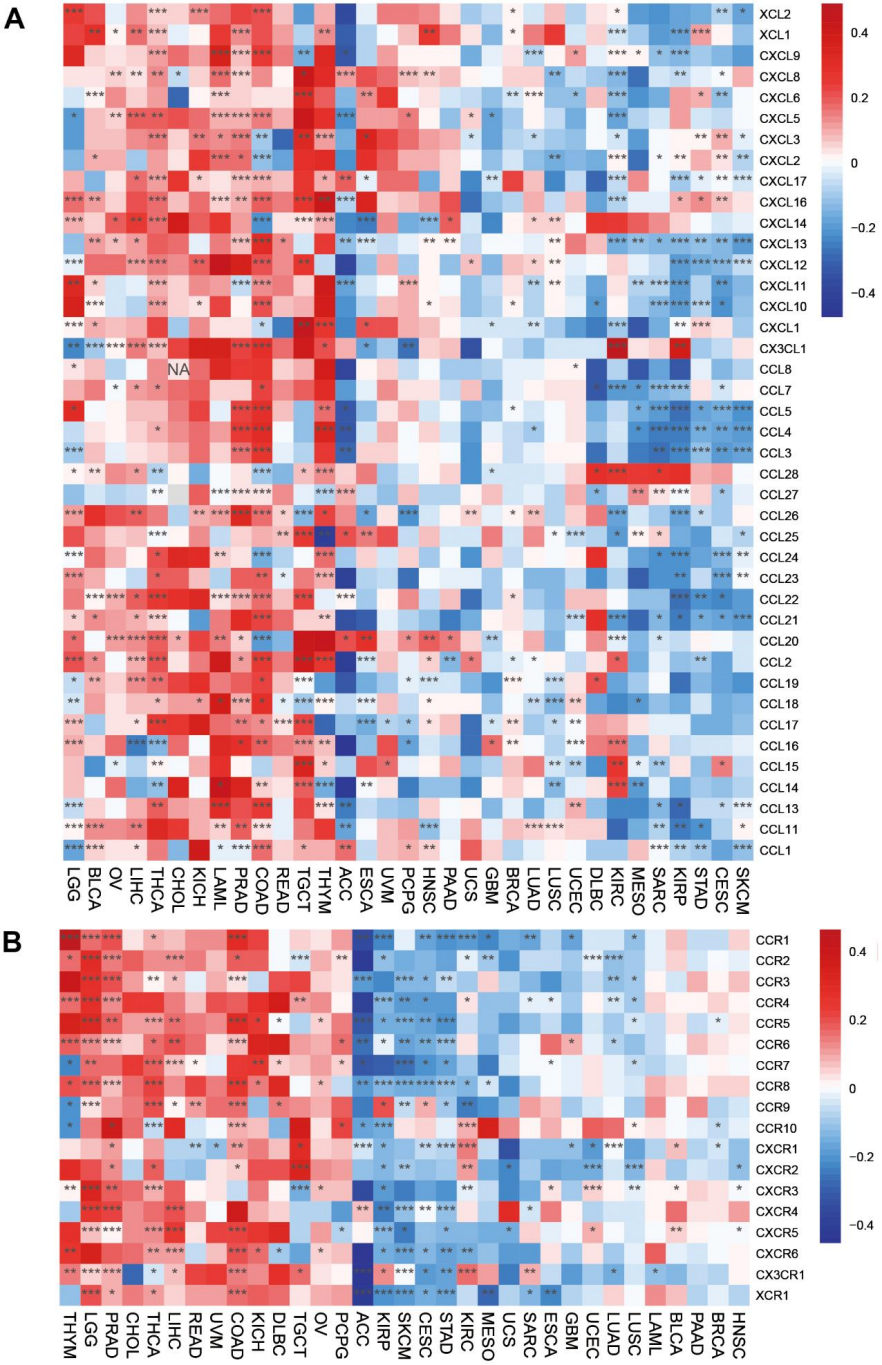
Associations between HOXC10 expression and (**A**) chemokine genes and theirs (**B**) receptors genes.

The directed migration of immune cells is a necessary condition for the occurrence and completion of immune response. Chemokines are a class of cytokines that control cell directed migration. Its function is mediated by chemokine receptors. The interaction between chemokines and their receptors controls the directional migration of various immune cells in the circulatory system and tissues and organs, so that they can go to the site of abnormal proliferation, destroy abnormal proliferation cells, and maintain the function of tissue cell balance. Our results suggested that chemokines and their receptors were positively associated with HOXC10 expression in LGG, THYM, PRAAD, CHOL, LIHC, READ, KICH, DLBC, and TGCT but were remarkably negatively associated with HOXC10 expression in ACC, SKCM, CESC, STAD, KIRP (Figure 6A, 6B).

### HOXC10 is upregulated in LUAD, especially in distant metastasis tissues, by bioinformatics analysis

**Figure 7:**
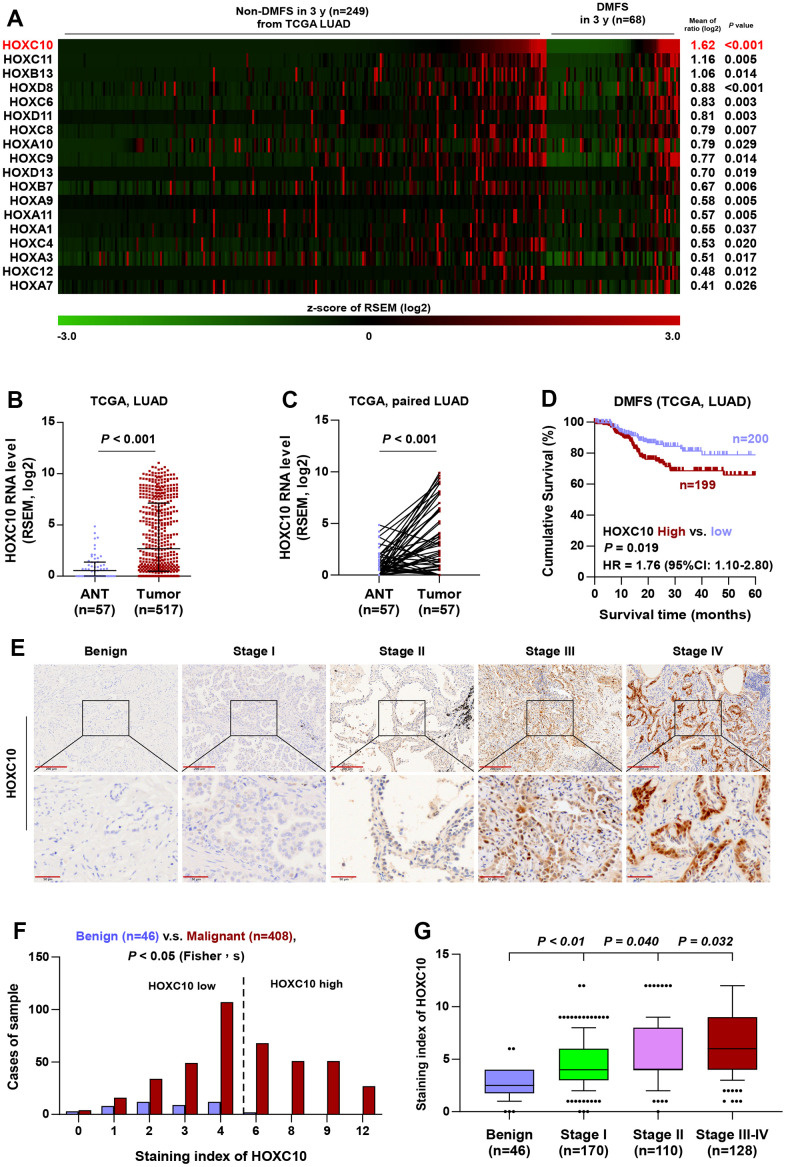
**HOXC10 is upregulated in LUAD.** (**A**) High expression of HOX genes in LUAD tissues with distant metastasis by analyzing the dataset of LUAD from TCGA. (**B**) Comparison of HOXC10 RNA expression between LUAD tissues and the adjacent normal tissues (ANT) in TCGA. (**C**) Comparison of HOXC10 RNA expression between LUAD tissues and the matched adjacent normal tissues (ANT) in TCGA. (**D**) Kaplan-Meier survival analysis of DMFS in LUAD patients with low HOXC10 expression versus high HOXC10 expression. (**E**) Representative images of HOXC10 immunostaining in 46 benign lung lesion and 408 LUAD tissues. (**F**) The number of lung tissues stratified by staining index of HOXC10. (**G**) Staining index of HOXC10 in different pathological stage of LUAD.

Since a large amount of literature supports the standpoint that HOX family genes are abnormally expressed in several types of human cancer and play vital roles in cancer progression and distant metastasis, the significance of HOX genes in LUAD seized our great momentum. By analyzing the mRNA expression of 39 HOX genes in the LUAD dataset from TCGA, we found that 18 HOX genes were significantly higher in LUAD tissues with distant metastasis in 3 years (n=249) compared with that without distant metastasis in 3 years (n=68), and HOXC10 was identified as the most upregulated HOX gene (Figure 7A). Therefore, we focused on the HOXC10 for further study. Moreover, the mRNA level of HOXC10 was significantly upregulated in the LUAD tissues (n=517) from TCGA compared with that in adjacent normal tissue (n=57) (Figure 7B). Similarly, HOXC10 was upregulated in LUAD tissue from TCGA (n=57) compared with matched adjacent normal tissue (Figure 7C). Importantly, the significance of HOXC10 expression on DMFS was analyzed in the LUAD dataset from TCGA, and LUAD patients with high expression of HOXC10 exhibited shorter DMFS compared with LUAD patients with the low level of HOXC10 (Figure 7D). Therefore, these findings from the LUAD dataset of TCGA indicated that HOXC10 is upregulated in LUAD tissues, especially with distant metastases, which predicted worse DMFS.

### Overexpression of HOXC10 in human LUAD samples indicates advanced progression and poor prognosis

To investigate the clinical significance of HOXC10 in LUAD, we further examined the expression of HOXC10 in a larger number of clinical samples, including 46 benign lung lesions and 408 LUAD tissues, by immunohistochemistry (IHC). As shown in Figure 7E–7G, HOXC10 was mainly expressed in the nucleus of cancer cells, and HOXC10 expression was dramatically higher in LUAD tissues than in benign lung lesions. HOXC10 upregulation was observed in 48.28% (197/408) LUAD tissues (Figure 7F and Table 1). Notably, the expression of HOXC10 was gradually increased with the clinical stage (Figure 7G).

**Table 1:** The relationship between HOXC10 expression level and clinical pathological characteristics in 408 patients with LUAD.

**Parameters**	**Number of cases**	**HOXC10 IHC expression**		***P*-values**
		**Low (n=211)**	**High (n=197)**	
Gender				
Female	165	88	77	0.590
Male	243	123	120	
Age				
<60	147	76	71	0.996
≥60	261	135	126	
Grade				
G1-G2	238	134	104	0.0283*
G3	170	77	93	
T classification (excluded other type)				
T1-2	322	167	155	0.904
T3-4	76	40	36	
N classification (excluded other type)				
N0	224	129	95	0.010*
N1-3	171	76	95	
M classification				
M0	370	199	171	0.009*
M1	38	12	26	
Stage				
I-II	280	158	122	0.005*
III-IV	128	53	75	

*IHC, Immunohistochemistry.

**Figure 8:**
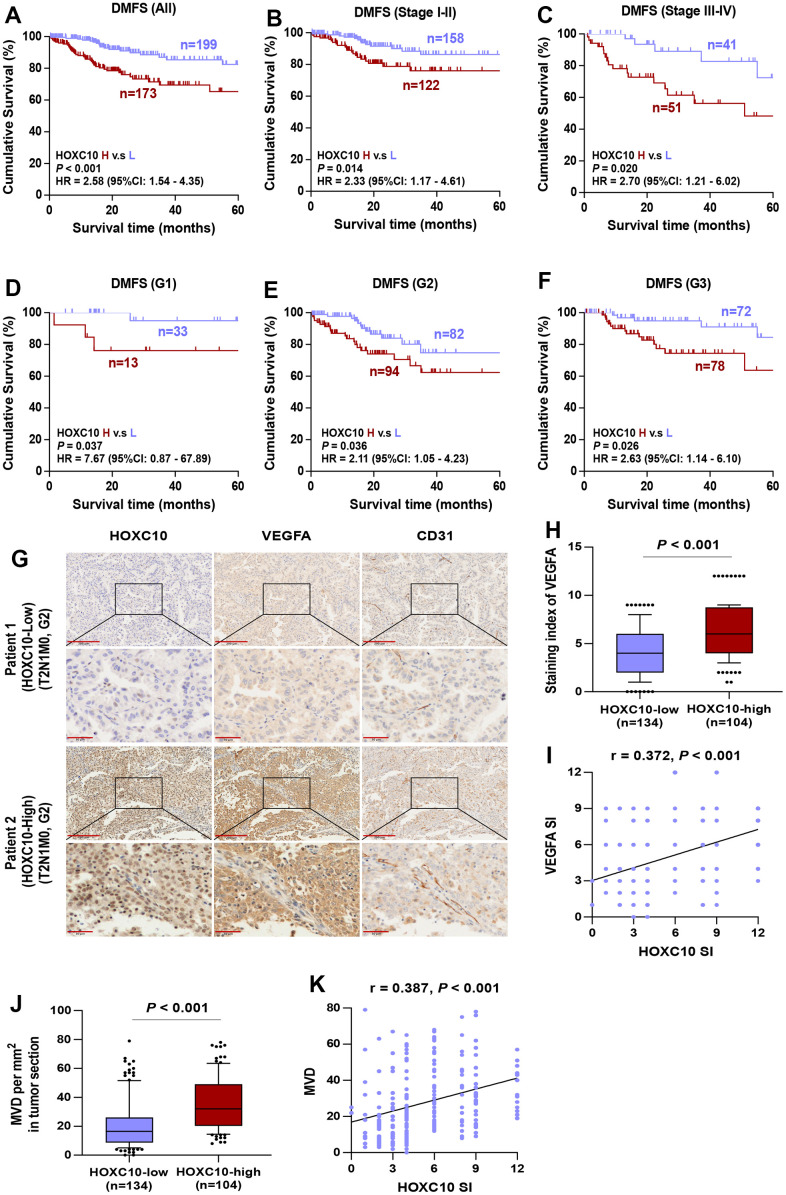
**High expression of HOXC10 in LUDA tissues represents a worse DMFS.** (**A**–**C**) Kaplan-Meier survival analysis of DMFS in all stage, stage I-II, and stage III-IV of LUAD patients with low HOXC10 expression versus high HOXC10 expression. (**D**–**F**) Kaplan-Meier survival analysis of DMFS in grade I, grade II, and grade III of LUAD patients with low HOXC10 expression versus high HOXC10 expression. (**G**) Representative IHC images of HOXC10, VEGFA and CD31 expression in LUAD tissues. (**H**) Staining index of VEGFA in HOXC10-low and HOXC10-high expression LUAD patients. Error bar represents the 10th-90th percentile. (**I**) The correlation between HOXC10 expression and VEGFA expression in LUAD samples. (**J**) MVD in HOXC10-low and HOXC10-high expression LUAD patients. (**K**) The correlation between HOXC10 expression and MVD in LUAD samples.

It is well known that the grade and stage of advanced tumors are significantly associated with the accelerated development of LUAD. Our statistical data indicated that elevated HOXC10 expression was positively correlated with pathological grade, N classification, M classification and clinical stage (Table 1). Furthermore, to analyze the effect of tumor stage and grade in LUAD patients with overexpressed HOXC10 on poor DMFS, LUAD patients were first stratified into different groups of Stage I to IV and Grade I to III. As shown in Figure 8A–8C, based on Kaplan-Meier survival analysis, high expression of HOXC10 exhibited poorer DMFS of LUAD patients with all Stage, Stage I-II, and Stage III-IV. Consistently, LUAD patient with high level of HOXC10 predicted shorter DMFS in the same pathological grade compared with low levels of HOXC10 (Figure 8D–8F). Collectively, these results demonstrated that the HOXC10 overexpression was significantly positively correlated with advanced progression and poor prognosis in LUAD patients.

### HOXC10 is an independent prognostic marker for DMFS of LUAD

To investigate whether HOXC10 is an independent prognostic value of DMFS in LUAD patients, univariate analysis of clinicopathological features and HOXC10 expression associated with DMFS in 408 LUAD patients was performed by Cox-regression analysis. LUAD patients with high levels of HOXC10 had a significantly higher risk of distant metastasis than patients with low levels of HOXC10 (hazard ratio [HR] = 2.426, 95% confidence interval [CI] = 1.417-4.153, *P*-value = 0.001). Similar results were shown for T classification (HR=1.887, 95%CI: 1.048-3.398, P=0.034), M classification (HR=31.258, 95%CI: 7.143-136.786, P<0.001), and clinical stage (HR=2.132, 95%CI: 1.264-3.598, P=0.005), while no significant correlation was found between N classification, age, gender, and pathological grade (Table 2). Multivariate analysis indicated that HOXC10 was an independent predictor for DMFS (HR=2.195, 955CI: 1.272-3.788, P=0.005, Table 2). Taken together, these studies suggest that HOXC10 is an independent prognostic marker for DMFS of LUAD.

**Table 2:** Univariate and multivariate Cox analysis of clinical parameters associated with DMFS in 408 LUAD patients.

**Parameters**	**Hazard ratio (95%CI)**	***P*-values**
**Univariate**		
Gender (male versus female)	0.818 (0.488-1.371)	0.446
Age (≥60 versus <60)	1.234 (0.707-2.152)	0.459
Grade (G3 versus G1-G2)	0.837 (0.492-1.423)	0.511
T classification (T3-4 versus T1-2)	1.887 (1.048-3.398)	0.034*
N classification (N1-3 versus N0)	1.371 (0.818-2.295)	0.231
M classification (M1 versus M0)	31.258 (7.143-136.786)	< 0.001*
Stage (III-IV versus I-II)	2.132 (1.264-3.598)	0.005*
HOXC10 expression (high versus low)	2.426 (1.417-4.153)	0.001*
**Multivariate**		
M classification (M1 versus M0)	14.669 (3.208-67.065)	0.001*
Stage (III-IV versus I-II)	1.816 (1.059-3.115)	0.030*
HOXC10 expression (high versus low)	2.195 (1.272-3.788)	0.005*

### Correlation of HOXC10 expression with the expression of angiogenic markers in LUAD

The growth of new capillaries in tumor tissue contributes to poor progression and metastasis, and it has been reported that HOXC10 is highly expressed in glioma and promotes angiogenesis [20]. To determine the possible association between HOXC10 and angiogenesis in LUAD patients, VEGFA and CD31 in LUAD patients were examined by IHC. Representative staining images immunohistochemical of HOXC10, VEGFA and CD31 in each group are shown in Figure 8G. HOXC10 expression was positively correlated with VEGFA expression (r = 0.372, *P* < 0.001) (Figure 8H, 8I). Moreover, MVD was efficiently enhanced in HOXC10-high tissues versus HOXC10-low tissues, as shown by CD31 staining (r = 0.387, *P* < 0.001) (Figure 8J, 8K). These data indicated that HOXC10 expression is positively correlated with angiogenic marker in LUAD.

### Correlation of HOXC10 expression with the number of CTC clusters in LUAD patients

**Figure 9:**
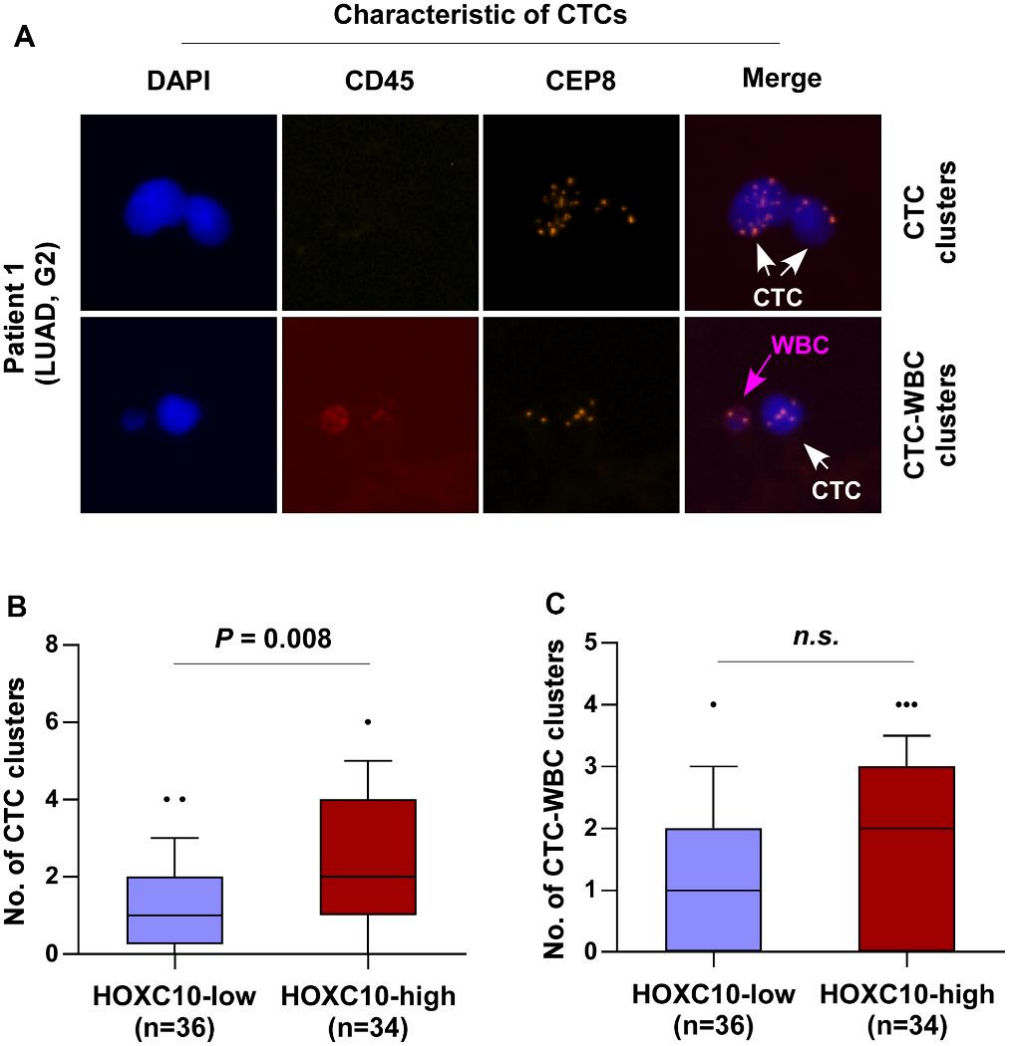
**HOXC10 expression is positively correlated with the number of CTC clusters in LUAD patients.** (**A**) Identification of CTC clusters and CTC-WBC clusters in LUAD patients by SE-iFISH platform. (**B**) Number of CTC clusters in HOXC10-low (n=36) and HOXC10-high expression (n=34) LUAD patients. (**C**) Number of CTC-WBC clusters in HOXC10-low (n=36) and HOXC10-high expression (n=34) LUAD patients.

The invasion of circulating tumor cells (CTCs) into peripheral blood represents a critical process to tumor metastasis, progression, and angiogenesis. Therefore, the clinical correlation of HOXC10 expression with the number of CTC clusters in LUAD patients was further analyzed. Immunofluorescence staining of CD45 and DAPI and iFISH with centromeres of chromosome 8 (CEP8) were used to identify CTCs in patients with LUAD. CD45-/CEP8 >2 cells were defined as CTCs, while CD45+/CEP8 = 2 cells were defined as white blood cell (WBC) (Figure 9A). We found that the number of CTC clusters in LUAD patients with high levels of HOXC10 (n= 34) was more than that in those with low levels of HOXC10 (n= 36) (Figure 9B). While the number of CTC-WBC clusters between them was no significant difference (Figure 9C). Our results indicated that HOXC10 expression levels is positively correlated with the number of CTC clusters in LUAD patients.

## DISCUSSION

The present study had several following findings: (1) HOXC10 was significantly up-regulated in most cancers including BRCA, CESC, DLBC, ESCA, GBM, KIRC, KIRP, LUAD, LUSC, PAAD, SARC, STAD, and THYM. However, the expression of HOXC10 was remarkably down-regulated in KICH, LAML, OV, PRAD, READ, SKCM, TGCT, THCA, UCEC. (2) Elevated HOXC10 was associated with poor prognosis in LGG, ACC, COAD, GBM, OV, BRCA, LIHC, LUSC, THCA, MESO, and PAAD but was negatively with survival prognosis in KIRC, KIRP, SKCM, and STAD. (3) HOXC10 involved in cancer via many different pathways and show dual roles. (4) HOXC10 may affect cancer occurrence and development via immune regulation. (5) HOXC10 could be a potential marker for aggression and metastasis in LUAD.

Our results indicated that HOXC10 was abnormal expression in most cancers, which emphasized the important roles of HOXC10 in cancers. Furthermore, the prognosis analysis also found that HOXC10 was associated with poor prognosis in LGG, COAD, ASS, LIHC, GBM, OV, THYM, STAD, MESO, CESC, UCS, and BLC but was associated with favorable prognosis in KIRC, KIRP and SKCM. These results indicated the dual roles of HOXC10. The reason could be tumor heterogeneity. The diversity of cells and genes present within the tumor, which may lead to different tumor responses to treatment and prognosis. Tumor heterogeneity is a major challenge in cancer treatment and research. Many common signaling pathways activation and inactivation were related to cancer progression. HOXC10 was positively enriched by most of signaling pathways. But for CESC, HNSC, and STAD, HOXC10 showed different correlations. Elevated HOXC10 was a risk factor for STAD while IL6/JAK/STAT3 and IL2/STAT5 signaling were negatively associated with HOXC10 in STAD. These results indicated that these pathways may be inactivated in STAD, which was consistent with previous study [21]. On the contrary, HOXC10 was positively associated with IL6/JAK/STAT3 and IL2/STAT5 signaling in glioma, and previous studies also indicated that these two signaling pathways were activated [22, 23]. We also evaluated the correlations of HOXC10 with immune infiltrations, and found that HOXC10 was positively associated with immune score in most cancers (LUAD, LUSC, TGCT, CESC, ESCA, PAAD, SARC, STAD, BRCA) except COAD. These results were consistent with prognosis analyses that high HOXC10 expression means poor prognosis in these cancers. Meanwhile, some immune cells, activated CD8 T cell, activated B cell, and macrophage were also positively expressed in these cancers. But oppositive results were found in COAD, KIRC, and KIRP. Immune score may not fully predict the prognosis for all types of cancers. Finally, we also analyzed the HOXC10 expression of some immune-related genes, which reflect different immune status.

We found abnormal expression HOXC10 was associated with poor DFI, and PFI in LUAD, and HOXC10 was also associated with TMB, MSI, ESTIMATE, stromal and immune score. Therefore, we chose the LUAD for further validation. To explore the specific function role, we investigated the roles of HOXC in LUAD. Metastasis is the major reason for high mortality rate and poor prognosis among patients with LUAD. HOX genes have pro-tumor and anti-tumor effects in different tumor tissues and can be a new target for cancer therapy. Therefore, the role and mechanism of HOX genes in LUAD is worth exploring. In this study, bioinformatics analysis showed that HOXC10 was the most highly expressed HOX genes in LUAD with metastasis than that without metastasis and HOXC10 levels were significantly higher in LUAD versus those in non-tumor tissues. Clinical specimens further confirmed that HOXC10 was overexpressed in LUAD, and positively correlated to the clinical stages. Importantly, the expression of HOXC10 is closely related to the level of angiogenic markers and the number of CTC clusters in clinical LUAD samples. Taken together, both the bioinformatics analysis and clinical data suggest that HOXC10 plays an important role in the metastasis of LUAD.

Angiogenesis, which refers to the shape of new blood vessels from the existing vascular system, occurs in many physiological and pathological processes, such as embryogenesis, wound healing, and cancer progression [24]. Anomalous angiogenesis is a necessary condition for new tumor growth, and plays a crucial role in the process of metastasis, which involves the cooperative effects of multiple molecules [25]. Two ordinary adhesion proteins play key impact in the process of angiogenesis: vascular endothelial growth factor A (VEGFA) and cluster of differentiation 31 (CD31). VEGFA is the most characteristic regulator of angiogenesis and metastatic growth in human cancer [26]. CD31 is a cell-cell adhesion molecule and a crucial role as MVD to evaluate the degree of tumor angiogenesis [27]. Numerous researches have demonstrated that HOX genes play a crucial impact in angiogenesis. Stephan et al. found that HOXB5 is an activator of angiogenesis that takes effect by upregulating angiopoietin2 [28]. Overexpressed HOXD3 promoted the migration, invasion, and angiogenesis of HCC cells [29]. Moreover, HOXC10 induces angiogenesis by upregulating the expression of VEGFA, and may be a potential antiangiogenic target for glioma [20]. In this study, we found that the expression of VEGFA and MVD were higher in LUAD clinical samples with HOXC10-high expression than that in samples with HOXC10-low expression, and the expression of VEGFA and MVD were positively correlated with the expression HOXC10. These data indicate that HOXC10 may be a potential pro-angiogenic factor of LUAD.

Circulating tumor cells (CTCs) has appealed considerable attention as a method of realizing the non-invasive, dynamic monitoring of cancer patients [30]. A series of recent studies have shown that CTCs circulate as seeds in the bloodstream at the initial stage of malignancy and may lead to a new metastasis [31–33]. CTCs in the blood of cancer patients can be detected as single CTC, CTC cluster, and CTC-WBC cluster, with the latter two characterizing a higher ability to metastasis [34, 35]. Although CTCs are extremely scarce compared to blood cells, they are considered as forerunner of metastasis in a variety of cancer types, including breast cancer, lung cancer, hepatocellular carcinoma, prostate cancer, et al. High level of CTC clusters had an increased risk of death, and the durability of CTC clusters during treatment was also a predictor of worsening outcome in breast cancer [36]. CTCs testing can better provide information for medical decision-making and improve patient outcomes in prostate cancer [37]. The number of CTCs is an independent prognostic factor for SCLC, and failure to reduce the number of CTCs below 50 after one cycle of chemotherapy indicates a poor prognosis [38]. In this study, we found that the number of CTC clusters in LUAD patients with high HOXC10 levels was more than that in LUAD patients with low HOXC10 levels, but there was no statistically significant difference in the number of CTC-WBC clusters between the two groups. The expression level of HOXC10 was positively correlated with the number of CTC clusters in LUAD patients. These results further validate that the high HOXC10 expression suggests a greater possibility of metastasis in LUAD.

Some study limitations should be addressed. One hand, the correlation of HOXC10 with immunes in cancers need to be verified *in vivo* and *in vitro*. On the other hand, more larger sample size is required for the association between HOXC10 expression and prognosis in LUAD.

In summary, our results demonstrated that aberrant expression happened in most cancers, which also affected the clinical prognosis in cancers. Abnormal expression HOXC10 involved in multiple signaling pathways, molecular features, and tumor microenvironment. The correlations of HOSC10 with immune score and immune-related genes showed that HOXC10 may promote or inhibit tumor occurrence and progression via regulating immune response. HOXC10 overexpression plays an important role in the aggression and metastasis in LUAD, which indicated a potential therapeutic target and an independent factor for the prognosis for LUAD patients.

## Supplementary Material

Supplementary Figures

Supplementary Tables
