# Supplementary material for: Pan-cancer analysis of homeodomain-containing gene C10 and its carcinogenesis in lung adenocarcinoma
Source: Aging (Albany NY). 2023 Dec 27;15(24):15243–66. doi: 10.18632/aging.205348 (PMC10781453; doi:10.18632/aging.205348)
Supplement: Supplementary Figures [file aging-15-205348-s001.pdf]

SUPPLEMENTARY FIGURES

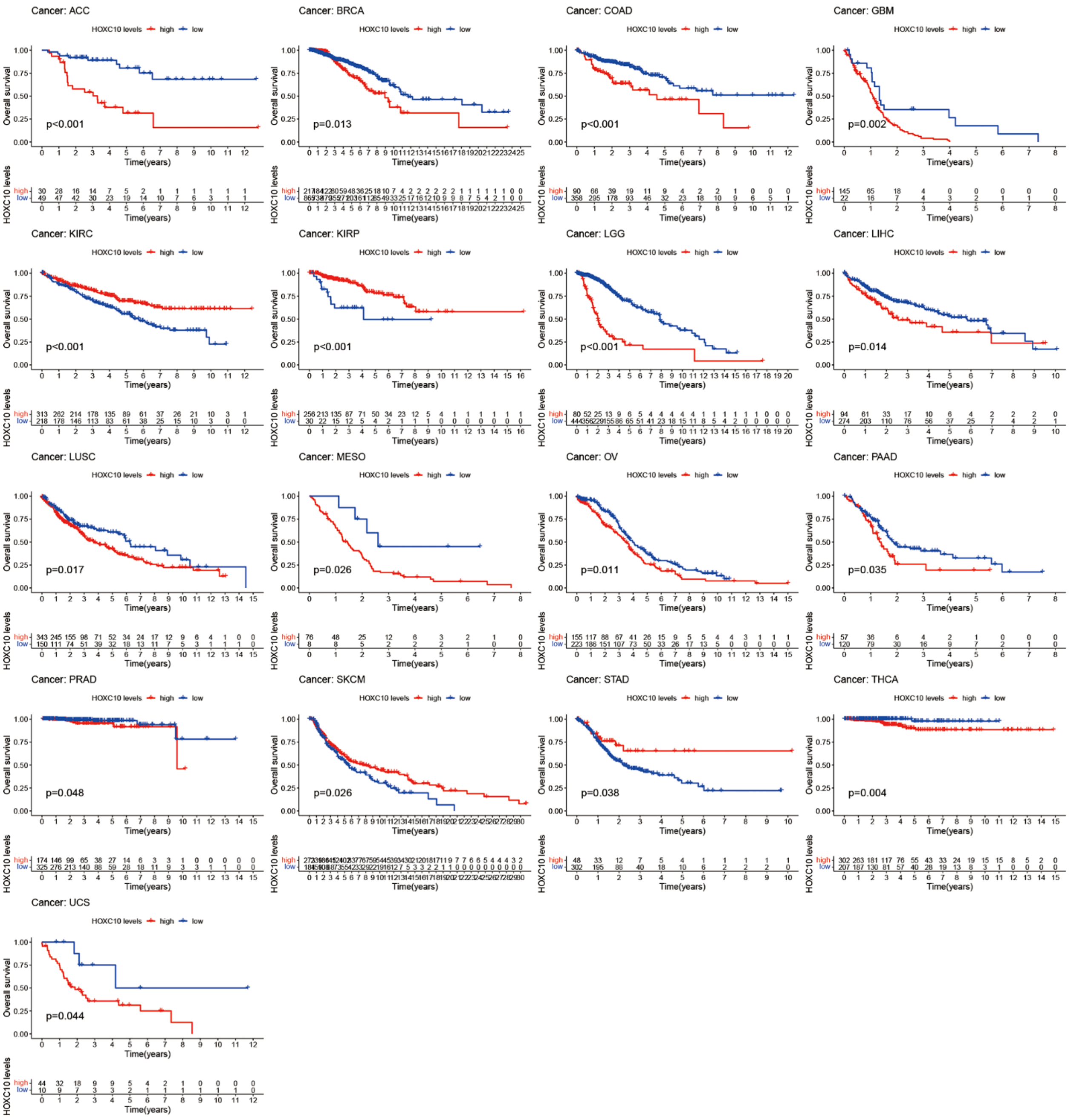

Supplementary Figure 1. Kaplan-Meier survival curves of correlations of HOXC expression with OS in cancers.

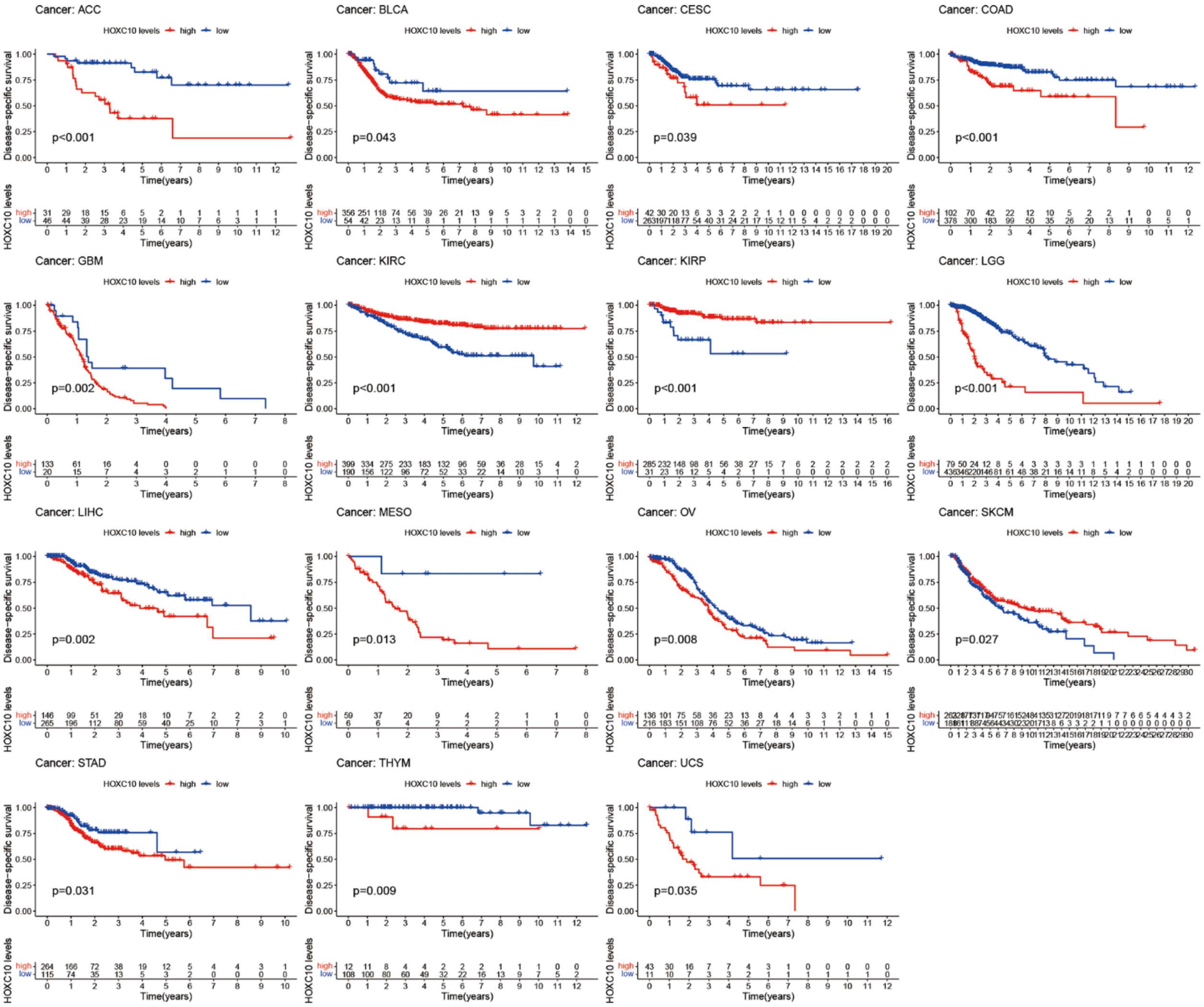

Supplementary Figure 2. Kaplan-Meier survival curves of correlations of HOXC expression with DSS in cancers.

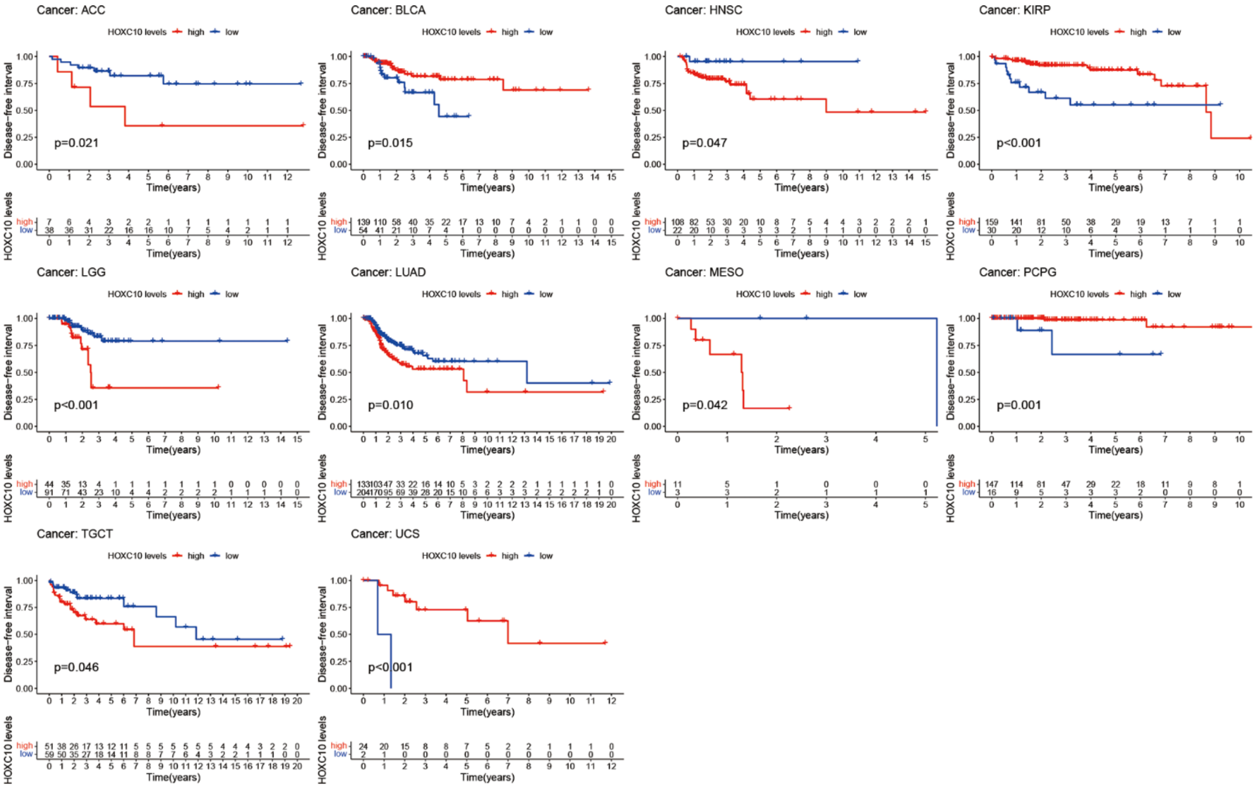

Supplementary Figure 3. Kaplan-Meier survival curves of correlations of HOXC expression with DFI in cancers.

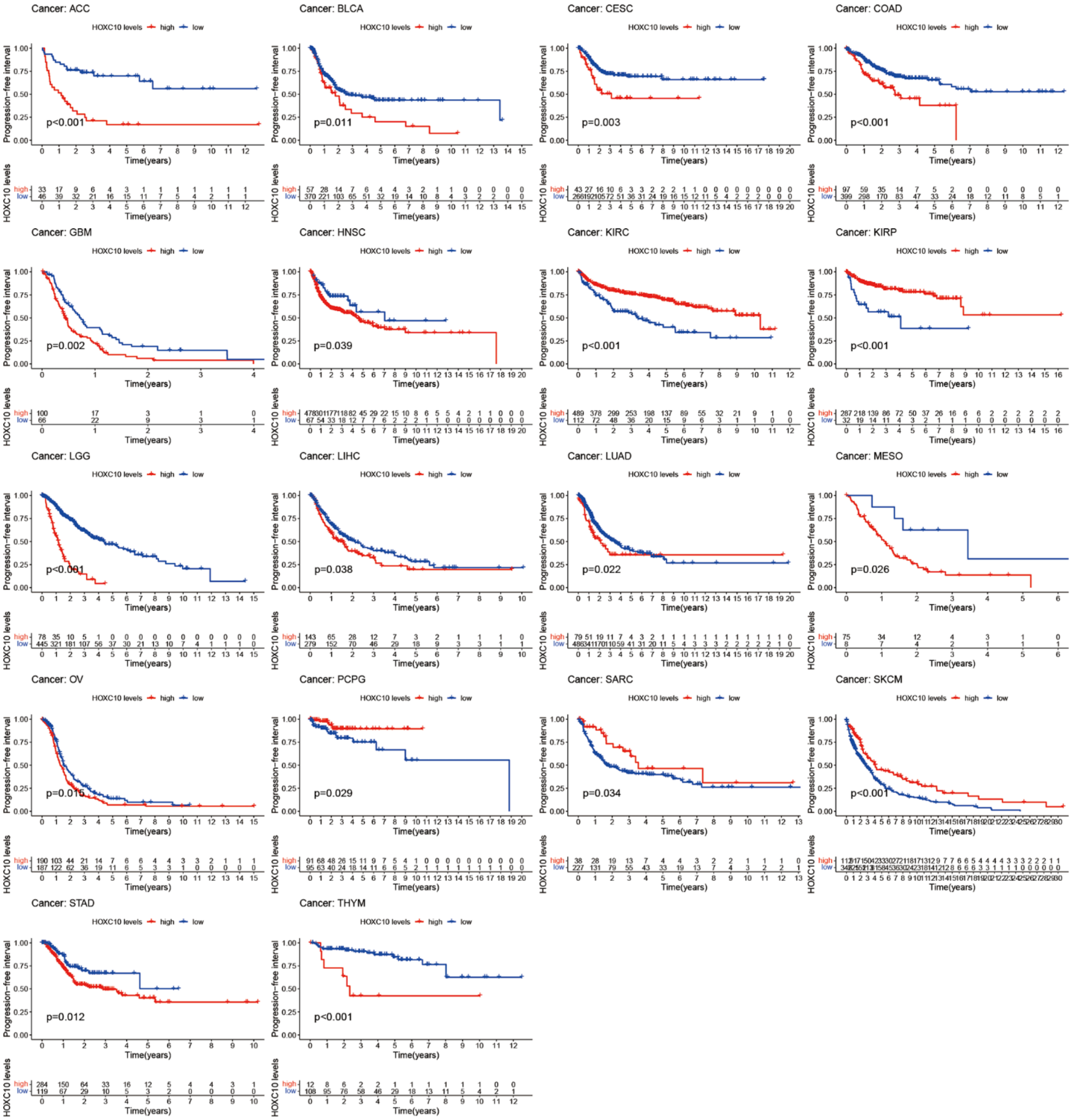

Supplementary Figure 4. Kaplan-Meier survival of curves correlations of HOXC expression with PFI in cancers.
