# Supplementary material for: Pan-cancer analysis of homeodomain-containing gene C10 and its carcinogenesis in lung adenocarcinoma
Source: Aging (Albany NY). 2023 Dec 27;15(24):15243–66. doi: 10.18632/aging.205348 (PMC10781453; doi:10.18632/aging.205348)
Supplement: Supplementary Tables [file aging-15-205348-s002.pdf]

SUPPLEMENTARY TABLES

Supplementary Table 1. The basic information of 46 patients with benign lung disease for HOXC10 in IHC analysis.

		Cases (n)	Percentage (%)
Gender	Male	35	76.1
	Female	11	23.9
Age	<60	34	73.9
	≥60	12	26.1
Type of diseases	Amyloidosis	1	2.2
	Fungal infection	2	4.3
	Hamartoma	3	6.5
	Hyperplasia	19	41.3
	Metaplasia	2	4.3
	Pneumonia	5	10.9
	Pulmonary bulla	5	10.9
	Sclerosing hemangioma	2	4.3
	Tuberculosis	7	15.2

Supplementary Table 2. The basic information of 408 patients with LUAD for HOXC10 IHC analysis.

		Cases (n)	Percentage (%)
Gender	Female	165	40.4
	Male	243	59.6
Age	<60	147	36.0
	≥60	261	64.0
Grade	G1	46	11.3
	G2	192	47.1
	G3	170	41.7
T classification	T1	112	27.5
	T2	210	51.5
	T3	52	12.7
	T4	24	5.9
	NA	10	2.5
N classification	N0	224	54.9
	N1	88	21.6
	N2	76	18.6
	N3	7	1.7
	NA	13	3.2
M classification	M0	370	90.7
	M1	38	9.3
Stage	Stage I	170	41.7
	Stage II	110	27.0
	Stage III	90	22.1
	Stage IV	38	9.3

*NA, Not available.
